# DNAJB3 attenuates metabolic stress and promotes glucose uptake by eliciting Glut4 translocation

**DOI:** 10.1038/s41598-019-41244-8

**Published:** 2019-03-18

**Authors:** Abdelilah Arredouani, Abdoulaye Diane, Namat Khattab, Ilham Bensmail, Imad Aoude, Mohamed Chikri, Ramzi Mohammad, Abdul Badi Abou-Samra, Mohammed Dehbi

**Affiliations:** 1 Qatar Biomedical Research Institute, Hamad Bin Khalifa University, Doha, Qatar; 2 Faculty of Medicine & Pharmacy, University Sidi Mohamed Ben Abdellah, Fes, Morocco; 3 The Interim Translational Research Institute, Hamad Medical Corporation, Doha, Qatar; 4 Karmanos Cancer Institute, Department Of Oncology, Wayne State University, Detroit, MI USA; 5 Qatar Metabolic Institute, Department of Internal Medicine, Hamad Medical Corporation, Doha, Qatar

## Abstract

Failure of the heat shock response is a key event that leads to insulin resistance and type 2 diabetes. We recently showed that DNAJB3 co-chaperone is downregulated in obese and diabetic patients and that physical exercise restores its normal expression with a significant improvement of the clinical outcomes. In 3T3-L1 adipocytes, DNAJB3 has a role in improving the sensitivity to insulin and glucose uptake. In co-immunoprecipitation assays, DNAJB3 interacts with both JNK1 and IKKβ kinases. However, the functional impact of such interaction on their activities has not been investigated. Here, we assessed the effect of DNAJB3 on the respective activity of JNK1 and IKKβ in cell-based assays. Using JNK1- and IKKβ-dependent luciferase reporters, we show a marked decrease in luciferase activity by DNAJB3 in response to PMA and TNF-α that was consistent with a decrease in the translocation of p65/NF-κB to the nucleus in response to LPS. Furthermore, TNF-α-mediated IL-6 promoter activation and endogenous mRNA expression are significantly abrogated by DNAJB3 both in 3T3-L1 and C2C12 cells. The ability of DNAJB3 to mitigate ER stress and oxidative stress was also investigated and our data show a significant improvement of both forms of stress. Finally, we examined the effect of overexpressing and knocking down the expression of DNAJB3 on glucose uptake in C2C12 as well as the molecular determinants. Accordingly, we provide evidence for a role of DNAJB3 in promoting both basal and insulin-stimulated glucose uptake. Our finding reveals also a novel role of DNAJB3 in eliciting Glut4 translocation to the plasma membrane. These results suggest a physiological role of DNAJB3 in mitigating metabolic stress and improving glucose homeostasis and could therefore represent a novel therapeutic target for type 2 diabetes.

## Introduction

Type 2 diabetes is a multifactorial metabolic disorder characterized by chronic hyperglycemia secondary to either increased insulin resistance (IR) in peripheral organs, progressive failure of the pancreatic islet β-cells or both^1^. The etiology of the disease is complex and involves an intricate interplay between genetic susceptibility and environmental factors, including sedentary lifestyles and obesity^2^. This latter is recognized as a major independent risk factor for type 2 diabetes through the development of IR^3^.

Metabolic stress is a prominent hallmark underlying both obesity and type 2 diabetes and it consists of a constellation of stress responses that are dysregulated in metabolically relevant sites. This include chronic metaflammation^4^, glucolipotoxicity^5^, increased oxidative stress^6^, mitochondrial dysfunction or biogenesis^7^, and persistent ER stress^8^ with the concomitant impairment of the anti-inflammatory response^9^, anti-oxidant defense system^10^ and the heat shock response (HSR)^11,12^. This metabolically toxic environment leads to a loss of homeostasis by activating several signaling pathways that abrogate the insulin action in insulin-responsive tissues^13^. The roles of c-Jun NH2-terminal kinase (JNK) stress kinase and the inhibitor of kappa B (IKKβ) inflammatory kinase in IR, β-cell function and type 2 diabetes are well established and as such, they emerged as attractive therapeutic targets for obesity-induced IR and type 2 diabetes. At the molecular level, both enzymes interfere with the insulin action by phosphorylating the inhibitory serine of the insulin receptor substrate (IRS) and thereby, converting it to a poor substrate for the activated insulin receptor^14,15^.

The HSR is a universal host-defence mechanism that plays a crucial role for cell survival under stressful conditions and this role is orchestrated by the immediate induction of a sub-set of highly conserved proteins called heat shock proteins (HSPs). HSPs were initially described as molecular chaperones involved in maintaining protein homeostasis by binding to misfolded and/or damaged proteins and assisting in their proper folding, disaggregation and remodelling^16^. Subsequent studies demonstrated that some of the HSPs (i.e. HSP-25 and HSP-72) act as natural inhibitors of JNK and IKKβ kinases and accordingly, they exhibit anti-apoptotic, anti-inflammatory and anti-oxidative stress properties^17–19^. In this regards, interventions that activate the HSR system are being intensively explored as alternative strategies to mitigate damages resulting from various stressful conditions including metabolic diseases^20–22^.

We recently reported the impaired expression of DNAJB3 cochaperone in adipose tissue biopsies isolated from obese non-diabetic^11^ and diabetic^23^ subjects, and that low levels of DNAJB3 were associated with enhanced metabolic stress^23^. More importantly, we showed that moderate physical exercise restores the normal expression of DNAJB3 with a significant improvement of the biochemical and clinical outcomes^11^. These findings suggest a potential protective role of DNAJB3 against obesity-induced IR and type 2 diabetes. DNAJB3; also known as Msj-1, is a member of the large DNAJ (HSP-40) family that was reported to play a role in male reproduction^11^. Its involvement in metabolic diseases began to be elucidated by our group. Accordingly, we demonstrated a role of DNAJB3 in improving insulin signaling and glucose uptake *in vitro* in 3T3-L1 adipocytes^23^. We also showed previously that DNAJB3 interacts with both JNK1 and IKKβ kinases in co-immunoprecipitation assays^11^. However, the functional consequence of such interactions remains unexplored.

In the current study, we used a series of functional assays to investigate the *in vitro* role of DNAJB3 in modulating metabolic stress and improving glucose uptake in HEK-293, C2C12 and 3T3-L1 cells. Using JNK1- and IKKβ-dependent luciferase reporters, we show a significant decrease in luciferase activity by DNAJB3 in response to phorbol myristate acetate (PMA) and tumor necrosis factor-α (TNF-α). Furthermore, TNF-α-mediated IL-6 promoter activation and the endogenous mRNA expression are significantly abrogated by DNAJB3. We finally provide evidence that DNAJB3 stimulates glucose uptake in C2C12 cells by eliciting Glut4 translocation to the plasma membrane.

Taken together, our results are suggestive of a physiological role of DNAJB3 in mitigating metabolic stress and regulating glucose homeostasis and insulin signaling and as such, it could represent a potential therapeutic target for metabolic diseases caused by increased IR.

## Materials and Methods

### Cell Culture

C2C12 myoblasts, 3T3-L1 preadipocytes, HEK-293 and HepG2 cells were all obtained from ATCC and maintained in DMEM supplemented with 10% FBS and 1% penicillin/streptomycin at 37 °C and 5% CO2. Differentiation of C2C12 myoblasts to myotubes was done by replacing FBS with 2% horse serum with a daily change of the media for 7 days. 3T3-L1 were differentiated from preadipocytes to adipocytes using isobutylmethylxanthine (IBMX), dexamethasone and insulin as we previously described^23^. All the cells were used before the 25^th^ passage.

### Plasmids and silencing RNA

pCMV-DNAJB3 and pCMV-HSPA1A plasmids were purchased from Origene (Origene Technologies, Inc., Rockvile, MD). They encode the human DNAJB3 and HSP-72, respectively. pCMV6 empty vector was used as a negative control. pHA-Glut4-GFP was a gift from Dr. MacGraw (Weill Cornell University, New York, NY) and consists of an exofacial HA epitope and a GFP tag located at the N-terminal and C-terminal of Glut4, respectively^24^. Reporter plasmids carrying firefly luciferase gene under the control of three copies of either wild type (3xwt-κB-Luc) or mutant (3xμκB-Luc) NF-κB binding site were described previously^23^. Reporter plasmid carrying the human IL-6 promoter (pIL6-Luc651)^25^, was obtained from Dr. Eickelberg (University of Colorado Denver, Aurora, CO). Reporter plasmid carrying seven copies of AP-1 binding site upstream of the firefly luciferase gene^26^, was obtained from Dr. Fahmi (Montreal University, Montreal, QC). ATF6-dependent reporter plasmid consisting of three copies of ATF6 response element upstream of the luciferase gene was purchased from Promega (Promega Corporation, Madison, WI). In all cases, Renilla Luciferase vector under the control of CMV promoter (pRL-CMV; Promega Corporation, Madison, WI) was used as internal control. Three different siRNA molecules specific for DNAJB3 and the scrambled siRNA were used to knockdown the expression of DNAJB3 in C2C12 (#SR406762; Origene Technologies, Inc., Rockville, MD).

### Transient transfections

Lipofectamine 3000 and RNAiMAX lipofectamine (Invitrogen, Carlsbad, CA) were used for transient DNA and siRNA transfection, respectively. All the functional assays were analyzed at least in triplicate and a minimum of three independent experiments.

### Luciferase assays

HEK-293 and C2C12 were transfected with 5 µg of the reporter plasmid and 10 µg of either pCMV-DNAJB3 or pCMV and then, plated on 96-well plates at 1.10^4^ cells/well followed by a 24-h incubation. Cells were then treated with 25 ng/ml of TNF-α (R&D Systems, Minneapolis, MN) or 5 µM PMA (Sigma Aldrich, St. Louis, MO) or 0.5 μg/ml Tunicamycin (Sigma Aldrich, St. Louis, MO) for 16 h and afterwards, harvested for luciferase assays using the Bright Glo Luciferase Assay kit (Promega, Madison, WI). Luciferase activity was measured either on Spark^®^ 10 M plate reader (Tecan, Männedorf, Switzerland) or Glomax multi detection plate reader (Promega, Madison, WI). Differences in transfection efficiency were normalized with pRL-CMV internal control.

### Measurement of gene expression by real-time PCR (RT-PCR)

Upon 48 h transfection of C2C12 and 3T3-L1 cells with 7.5 µg of either pCMV-DNAJB3 or pCMV and stimulation with either the vehicle, 3 h incubation with 50 ng/ml TNF-α (R&D Systems, Minneapolis, MN) or an overnight incubation with 0.5μg/ml Tunicamycin (Sigma Aldrich, St. Louis, MO), total RNA was extracted using RNeasy Plus Universal Mini Kit (Qiagen, Hilden, Germany). It was then converted to cDNA using MMLV Reverse Transcriptase Kit (Invitrogen, Carlsband, CA) and analyzed by RT-PCR on QuantStudio 6 Flex system (ThermoFisher, Waltham, MA), using SYBR Green. Relative expression was calculated by the comparative ΔΔCT method. The sequences of the primers used in this study are listed in Table 1.

**Table 1:** Primer list and sequences.

Gene	Forward	Reverse
DNAJB3	5′-AGGGGCTGTACCCTTCTCTA-3	5′-AGTTTCCTGGAGAACCGAAG-3′
IL-6	5′-GATGGATGCTACCAAACTGG-3′	5′-TGAAGGACTCTGGCTTTGTC-3′
Glut4	5′-TGGGTCCTTACGTCTTCCTT-3′	5′-GCTGAGATCTGGTCAAACGT-3′
Glut1	5′-TACCAAAGTTATCCGGCAGC-3′	5′-CTCTGAGTCTCAATGGCCAC-3′
Actin	5′-AAGAGCTATGAGCTGCCTGA-3′	5′- GATGCCACAGGATTCCATAC-3′
GAPDH	5′-CTGGAGAAACCTGCCAAGTA-3′	5′-AGTGGGAGTTGCTGTTGAAG-3′
SOD1	5′-GAGAGGCATGTTGGAGACCT-3′	5′-CCACCTTTGCCCAAGTCATC-3′
Catalase	5′-AGGAGGCAGAAACTTTCCCA-3′	5′-GGCCCTGAAGCATTTTGTCA-3′
GPX1	5′-ATCAGTTCGGACACCAGGAG-3′	5′-GATGTACTTGGGGTCGGTCA-3′
XBP1	5′-TCCCCAGAACATCTTCCCAT-3′	5′-ACATGACAGGGTCCAACTTG-3′
GRP78	5′-AATTTCTGCCATGGTTCTCA-3′	5′-AGCATCTTTGGTTGCTTGTC-3′

### Preparation of the whole protein extracts, nuclear and cytoplasmic extracts

Whole protein extracts were prepared from C2C12 and HEK-293 cells by resuspending cells in RIPA buffer (50 mM Tris-HCl pH 7.5, 150 mM NaCl, 1% Triton X-100, 0.5% Na-Deoxycholate, 0.1% SDS and Protease Inhibitor Cocktail (Sigma Aldrich, St. Louis, MO) and incubating the homogenates for 30 min at 4 °C. The extracts were then centrifuged at 13,000 rpm for 20 min and the supernatants were collected. The preparation of nuclear and cytoplasmic extracts from C2C12 myoblasts was carried out by using the ReadyPrep™ Cytoplasmic/Nuclear Extraction Kit (Bio-Rad, Hercules, CA) according to the manufacturer’s protocol. Protein concentration was determined by Bradford assay (Biorad) at 595 nm using γ-Globulin (Bio-Rad, Hercules, CA) as standard. Proteins were aliquoted and stored at −80 °C until assayed.

### Western blot analysis

Whole protein extracts prepared from HEK-293 cells transfected with 10 µg of either pCMV-DNAJB3, pCMV or pCMV-HDAC4 vectors were used to monitor the changes in the phosphorylation levels of JNK (P-JNK) in response to 5 µM PMA stimulation by western blot essentially as we described previously^23^. The expression of DNAJB3 and HSP-72 in whole cell extracts prepared myoblasts and myotubes was also performed by western blot using anti-DNAJB3 (Proteintech Group, Inc., Chicago, IL) and anti-HSP-72 (ENZO Life Sciences, Inc., Farmingdale, NY) antibodies. The endogenous expression of Glut4 in C2C12 overexpressing DNAJB3 (or control vector) was monitored by western bot using anti-Glut4 antibody (Abcam, Cambridge, UK). Nuclear translocation of p65 NF-κB in C2C12 transfected with DNAJB3 or pCMV after LPS/TNF-α stimulation was carried out on cytoplasmic and nuclear fractions by western blot using anti-p65 antibody (Cell Signaling Technology, Inc., Danvers, MA). Anti-GRP78 antibody (Cell Signaling Technology, Inc., Danvers, MA) was used to monitor the expression of GRP78 protein in response to Tunicamycin treatment using whole cell extracts from C2C12 transfected with DNAJB3 or pCMV. GAPDH, β-Actin (Cell Signaling Technology, Inc., Danvers, MA) and γ-Tubulin (Acam, Cambridge, UK) were used as internal controls as indicated in the figure legends.

### Glucose uptake assay

Cells were grown in 100 mm petri dishes until they reached 80% confluence and then, transfected with 7.5 µg of either pCMV-DNAJB3 or pCMV or 10 nM of DNAJB3-siRNA. The next day, they were plated on 96-well plates at 1.10^4^ cells/well and then used to monitor glucose uptake using the fluorescent D-glucose analog (2-NBDG) (Cayman, Ann Arbor, MI) as we described previously^23^, except that cells were glucose-starved for overnight while HepG2 cells were starved only for 3 h. After washes, the retained fluorescence was measured respectively at excitation and emission wavelengths of 485 nm and 535 nm with FLUOstar Omega microplate reader (BMG Labtech, Ortenberg, Germany).

### Monitoring Glut4 translocation by immunofluorescence and confocal microscopy

C2C12 transfected with 5 µg of pHA-Glut4-GFP plasmid and 10 µg of either pCMV-DNAJB3 or pCMV were plated on glass-bottom dishes. After stimulation with 100 nM of Insulin (Sigma Aldrich, St. Louis, MO), they were fixed with 4% paraformaldehyde without permeabilization and subjected to HA staining using a rabbit monoclonal anti-HA antibody (Rockland, Limerick, PA) followed by Alexa Fluor 594-conjugated goat anti-rabbit IgG (Abcam, Cambridge, UK). The Alexa Fluor 595/GFP ratio was determined by quantitative fluorescence microscopy as described previously^27^. To avoid cell selection bias fields, cells expressing the HA–Glut4-eGFP were randomly chosen in the GFP channel blinded to the expression of HA–Glut4-GFP on the plasma membrane (Alexa Fluor 594 channel). Images were collected in both GFP and Alexa Fluor 594 channels. To optimize the dynamic range of the assay, exposure times for the channels were independently set to maximize the signal while minimizing the number of cells with expression levels above saturation. Once set for each channel, all images in that channel were collected at the same exposure. The fluorescence intensities of GFP and Alexa 594 were quantified at the single-cell level. Mock- transfected cells were used in parallel to correct for fluorescence resulting from non-specific binding of the primary and/or secondary antibodies.

### Statistical analysis

Results are presented as means ± SEM and were plotted using GraphPad (Prism v7, La Jolla, CA). We used one-way ANOVA for comparison of the groups with post-hoc Tukey’s test or the Student t test, as appropriate. A P-value < 0.05 was considered statistically significant.

## Results

### Overexpression of DNAJB3 reduces JNK1 phosphorylation and abolishes its activity in a JNK1-dependent luciferase assay

**Figure 1:**
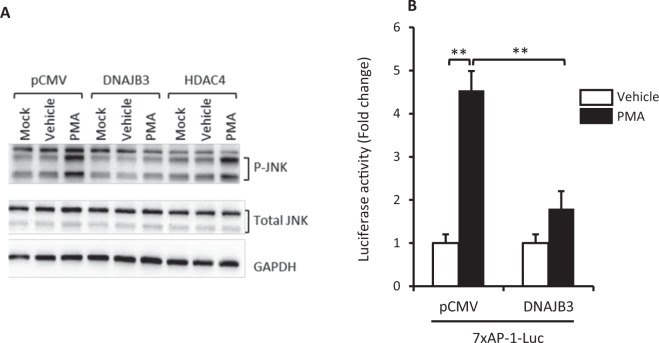
DNAJB3 acts as natural inhibitor of JNK1 stress kinase. (**A**) Transient overexpression of DNAJB3 in HEK-293 cells prevents the phosphorylation of JNK (P-JNK) in response to phorbol myristate acetate (PMA) as compared to pCMV and pCMV-HDAC4. Total JNK and GAPDH were used as internal controls to monitor for protein loading differences. After determining the levels of P-JNK, the same membrane was stripped and probed with antibody against total JNK. antibody. Full-length blots are displayed in Supplementary Fig. S1. (**B**) DNAJB3 abrogates PMA-mediated AP-1-dependent transactivation in luciferase assays. DMSO was used at 0.25% as a vehicle. **P < 0.01; NS: Not significant.

We previously reported that JNK is part of a multicomponent complex that interacts with DNAJB3 in immunoprecipitation assays^11^. Our current data (Fig. 1A) and previous data^23^ show a clear reduction in the levels of P-JNK1 in cells overexpressing DNAJB3 in response to PMA stimulation. Since JNK is also known to modulate gene expression via AP-1 cis-regulatory element^28^, we investigated the impact of DNAJB3 on JNK activity using a functional assay. To this end, HEK-293 cells were co-transfected with p7xAP-1-Luc with either pCMV-DNAJB3 expression vector or pCMV vector and the luciferase activity was monitored after PMA treatment. Data shown in Fig. 1B indicate that PMA treatment triggers 4–5 fold increase in luciferase activity as compared to the vehicle. In cells overexpressing DNAJB3, the luciferase activity was significantly reduced (P < 0.001), confirming thus the observed reduced levels in P-JNK triggered by DNAJB3.

### DNAJB3 abrogates PMA and TNF-α-mediated IKKβ activation

**Figure 2:**
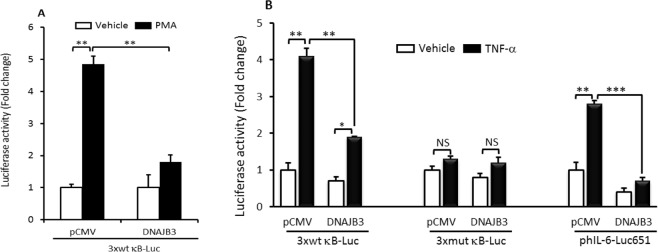
DNAJB3 acts as natural inhibitor of IKKβ inflammatory kinase. (**A**) Transient overexpression of DNAJB3 in C2C12 cells prevents the activation of κB-dependent transactivation in response to phorbol myristate acetate (PMA) in luciferase assays. (**B**) DNAJB3 abrogates also TNF-α-mediated both κB- and IL-6 promoter-dependent luciferase activation. DMSO at 0.25% and PBS were used as vehicles for PMA and TNF-α treatments, respectively. **P < 0.01; ***P < 0.001.

IKKβ has also been shown to interact with DNAJB3^11^, however, the functional consequence of such interaction was not explored. In this study, we interrogated whether DNAJB3 could interfere with NF-κB activation using our previously established κb-dependent luciferase system^23^. To this end, cells were co-transfected with p3xwtκB-Luc reporter and either pCMV-DNAJB3 or the pCMV and subsequently, they were stimulated with 5 µM of PMA or 25 nM TNF-α and then, the luciferase activity was monitored. As shown in Fig. 2A, there was a 5-fold increase of luciferase activity in response to PMA treatment in cells cotransfected with pCMV. In cells overexpressing DNAJB3, the luciferase activity was markedly reduced (P < 0.01; Fig. 2A). A similar increase in luciferase activity following stimulation with TNF-α in cells transfected with p3xwtκB-Luc construct but not with p3xmutκB-Luc construct (P < 0.01; Fig. 2B). DNAJB3 overexpression abolished the κB-dependent luciferase activity triggered by TNF-α- (P < 0.001; Fig. 2B). These findings prompted us to assess the effect of DNAJB3 in controlling NF-κB activity using a physiologically relevant context such as the IL-6 promoter whose activity is in part, regulated by NF-κB^25^. As expected, DNAJB3 reduced significantly the activity of IL-6 promoter following TNF-α stimulation (P < 0.001; Fig. 2B). Together, these data indicate that DNAJB3 acts upstream of the NF-κB signaling pathway and support our previous findings showing IKKβ as an interacting partner of DNAJB3.

### DNAJB3 modulate the expression of the IL-6 mRNA in response to TNF-α

**Figure 3:**
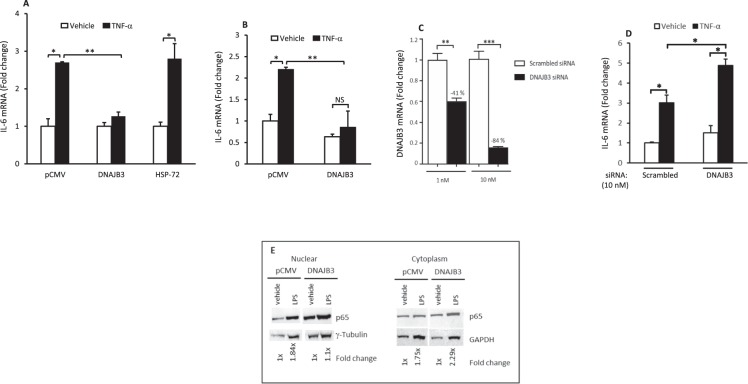
DNAJB3 is involved in TNF-α-mediated IL-6 mRNA expression. (**A**,**B**) Overexpression of DNAJB3 reduces significantly the endogenous expression of IL-16 mRNA in response to TNF-α both in C2C12 (**A**) and 3T3-L1 adipocytes (**B**). (**C**) Silencing the expression of DNAJB3 in C2C12 myoblast with specific siRNA reduced significantly the expression of DNAJB3 mRNA in a dose dependent-manner. GAPDH gene was used as a reference for normalization. (**D**) Knocking down the expression of DNAJB3 in C2C12 myoblasts resulted in a significant increase in TNF-α-mediated IL-6 mRNA expression. E: Overexpression of DNAJB3 in C2C12 myoblasts reduces the translocation of p65 NF-κB to the nucleus in response to LPS treatment (1 μg/ml for 3 h). Full-length blots are displayed in Supplementary Fig. S2. PBS was used as a vehicle. *P < 0.05; **P < 0.01; ***P < 0.001; NS: Not significant.

To complement these luciferase assays, we investigated the effect of DNAJB3 on the endogenous expression of IL-6 mRNA by RT-PCR. Results displayed in Fig. 3A,B show a 2- to 2.5-fold increase in IL-6 mRNA expression upon stimulation with TNF-α as compared to the vehicle (P < 0.05) in C2C12 and 3T3-L1 cells, respectively. Overexpression of DNAJB3 caused a significant reduction of IL-6 mRNA expression following TNF-α stimulation as compared to pCMV (P < 0.01) both in C2C12 myoblasts (Fig. 3A) and 3T3-L1 adipocytes (Fig. 3B). Under the same conditions, we failed to demonstrate a role of HSP-2 in preventing the response of IL-6 mRNA expression to TNF-α (Fig. 3A). To corroborate these findings, we silenced the expression of DNAJB3 using specific siRNA. We first determined the efficiency and specificity of these siRNA to abrogate the endogenous expression of DNAJB3 by RT-PCR in C2C12 myoblasts. As expected, transfection of cells with 10 nM DNAJB3 siRNA reduced the expression of DNAJB3 mRNA by 84% as compared to control siRNA (P < 0.0001; Fig. 3C). The response of IL-6 mRNA expression to TNF-α under the conditions where DNAJB3 expression is silenced was investigated in C2C12 myoblasts and the finding is displayed in Fig. 3D. As shown, there was a slight increase in both basal and TNF-α induced IL-6 mRNA expression as compared to scrambled siRNA (P < 0.05).

On the light of these results, we decided to determine the effect of DNAJB3 on the translocation of NF-κB in response to inflammatory inducers in C2C12 myoblasts. Accordingly, we used western blot to monitor the translocation of p65 subunit to the nucleus in response to LPS stimulation and the data are displayed in Fig. 3E. As shown, there was a decrease of approximately 60% of p65 in the nucleus in response to LPS when DNAJB3 is overexpressed as compared to pCMV. In the cytoplasmic fraction, LPS triggered an increase in p65 levels in both pCMV and DNAJB3 transfected cells by 1.75- and 2.29-fold, respectively (Fig. 3E). These findings support further the anti-inflammatory property of DNAJB3.

### DNAJB3 has a positive effect in alleviating ER stress and enhancing the oxidative stress scavenging system

**Figure 4:**
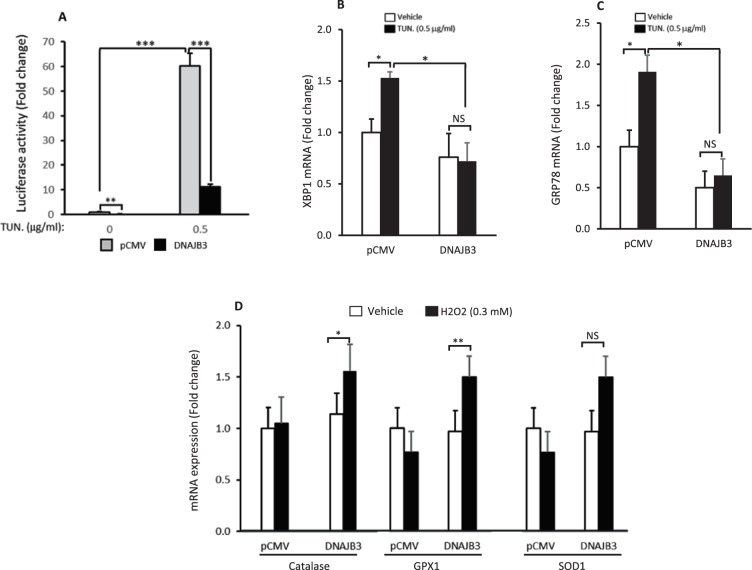
Overexpression of DNAJB3 alleviates basal ER stress and enhances the oxidative stress scavenging system. (**A**) Transient overexpression of DNAJB3 in C2C12myoblasts cells reduces significantly the basal activity of ATF6 in Luciferase assays. B-C: DNAJB3 abrogates also Tunicamycin-mediated mRNA expression of both XBP-1 (**B**) and GRP78 (**C**). (**D**) Overexpression of DNAJB3 in C2C12 cells stimulates the endogenous mRNA expression of Catalase and Glutathione peroxidase 1 (GPX1) genes in response to 300 μM H_2_O_2_ treatment for 3 h. DMSO at 0.25% and PBS were used as vehicles for Tunicamycin and TNF-α treatments, respectively. *P < 0.05; **P < 0.01; ***P < 0.001.

The contribution of persistent ER stress and enhanced oxidative stress to the pathogenesis of IR and T2D promoted us to assess the effect of DNAJB3 on mitigating ER stress and oxidative stress. For this purpose, we used a luciferase reporter assay driven by multiple copies of ATF-6 transcription factor; the master transcription factor involved in the activation of ER stress^29^. C2C12 myoblasts were cotransfected with ATF-6 reporter and either pCMV-DNAJB3 or pCMV and then stimulated for overnight with 0.5μg/ml of Tunicamycin. As shown in Fig. 4A, DNAJB3 reduces significantly (P < 0.01) the luciferase activity at both basal level and following Tunicamycin stimulation. This data is suggestive of a role of DNAJB3 in alleviating the ER stress. To complement this finding, we examined the effect of DNAJB3 on the endogenous expression of representative markers of ER stress; namely GRP78 and XPB1 in response to Tunicamycin. Data displayed in Fig. 4B,C show a significant decrease in both XBP1 and GRP78 mRNA levels in DNAJB-transfected cells upon Tunicamycin treatment (P < 0.05).

We then assessed the ability of DNAJB3 to modulate the expression of key representative antioxidant enzymes in response to oxidative stress. Results displayed in Fig. 4D indicate a significant increase in the expression of Catalase (P < 0.05) and GPX1 (P < 0.01) in response to H_2_O_2_ as compared to pCMV.

Taken together, these findings indicate that DNAJB3 has a positive effect in reducing ER stress and enhancing the antioxidant defense system.

### DNAJB3 enhances basal and insulin-stimulated glucose uptake

**Figure 5:**
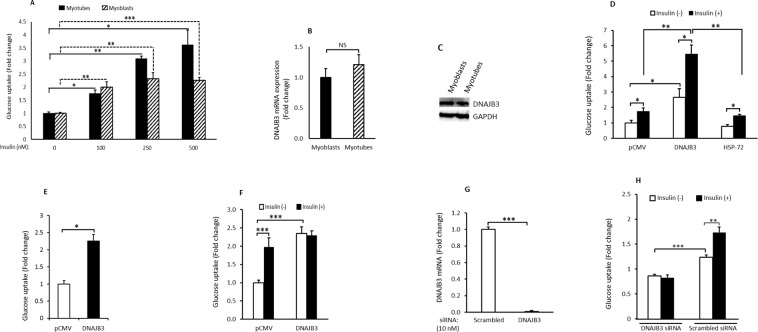
DNAJB3 promotes glucose uptake in C2C12 cells. (**A**) Dose response effect of insulin on glucose uptake in myoblasts (dashed box) and myotubes (black box). (**B**) RT-PCR data showing the expression of DNAJB3 in myoblasts and myotubes. GAPDH was used as a control. (**C**) Western blot showing the expression pattern of DNAJB3 myoblasts and myotubes. GAPDH was used as a reference for normalization. (**D**) DNAJB3 promotes both basal and insulin-stimulated glucose uptake in myoblasts as compared to pCMV. HSP-72 has no role on glucose uptake. (**E**,**F**) Overexpression of DNAJB3 promotes glucose uptake in 3T3-L1 adipocytes (**E**) and HepG2 cells (**F**). (**G**) Silencing the expression of DNAJB3 with 10 nM of specific siRNA blunted the expression of DNAJB3 mRNA in C2C12 myoblasts. Actin gene was used as a reference control. H: Knocking down the expression of DNAJB3 expression with specific siRNA abrogated both basal and insulin-stimulated glucose uptake in C2C12 cells. *P < 0.05; **P < 0.01; ***P < 0.001.

In 3T3-L1 adipocytes and HEK-293 cells, we previously showed that DNAJB3 promotes glucose uptake^23^. In this study, we investigated whether overexpression of DNAJB3 in C2C12 could enhance glucose uptake. We initially compared the glucose uptake in differentiated (myotubes) and undifferentiated (myoblasts) C2C12 in response to insulin and the data shown in Fig. 5A revealed a subtle difference in insulin-stimulated glucose uptake between myoblasts and myotubes. We then determined the expression levels of DNAJB3 mRNA and protein before and after differentiation of C2C12 cells. Results displayed in Fig. 4 indicate a modest change in the levels of DNAJB3 mRNA (Fig. 5B) and protein (Fig. 5C) following differentiation of C2C12 from myoblasts to myotubes. Based on these observations, we decided to use C2C12 myoblasts as a surrogate cellular model to study the effect of DNAJB3 on glucose uptake in response to insulin. In transfected myoblasts, DNAJB3 triggers a significant increase in basal glucose uptake as compared to pCMV (P < 0.05; Fig. 5D). In response to insulin stimulation, we observed a further increase in glucose uptake in cells overexpressing DNAJB3 as compared to pCMV and HSP-72 (P < 0.01; Fig. 5D). In 3T3-L1 adipocytes, a significant increase was also observed in cells overexpressing DNAJB3 in response to insulin stimulation but it was less pronounced than in C2C12 cells (P < 0.05; Fig. 5E). The effect of DNAJB3 on glucose uptake in HepG2 cells was also investigated in this study and the data are displayed in Fig. 5F. As shown, DNAJB3 triggers a marked increase in basal glucose uptake in HepG2 cells as compared to pCMV (P < 0.001; Fig. 5F). Stimulation with insulin did not show any additive effect on glucose uptake in cells overexpressing DNAJB3 while in pCMV transfected cells, a 2-fold increase in glucose uptake was observed (P < 0.001; Fig. 5F).

To further validate the direct role of DNAJB3 in promoting glucose uptake, we silenced the expression of DNAJB3 using siRNA. As shown in Fig. 5G, 10 nM of DNAJB3 siRNA blunted the expression of DNAJB3 mRNA in C2C12 myoblasts (P < 0.0001). We then examined the effect of knocking down the expression of DNAJB3 on glucose uptake and the result is displayed in Fig. 5H. As shown, knocking down the expression of DNAJB3 reduced significantly both basal (P < 0.0001) and insulin stimulated (P < 0.01) glucose uptake as compared with scrambled siRNA. These results suggest an important role of DNAJB3 in enhancing glucose uptake in various metabolically relevant cells.

### Overexpression of DNAJB3 elicits Glut4 translocation to the plasma membrane in C2C12 cells

**Figure 6:**
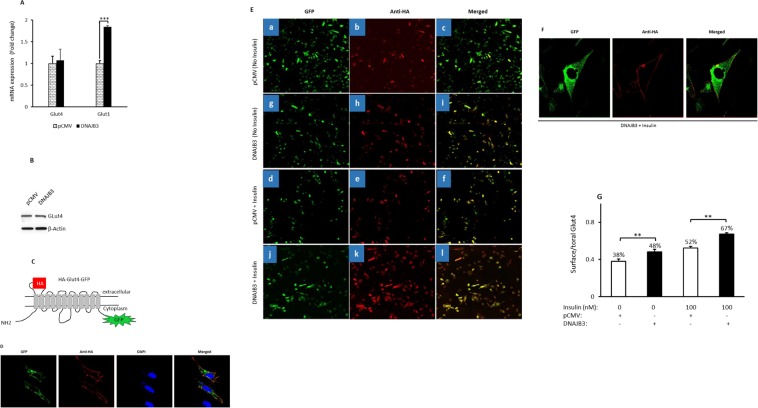
DNAJB3 elicits the translocation of Glut4 transporter to the plasma membrane in C2C12 cells without changing its expression. (**A**) Effect of DNAJB3 on the endogenous expression of Glut1 and Glut4 mRNA. (**B**) Effect of DNAJB3 on Glut4 protein expression. Full-length blots are displayed in Supplementary Fig. S3. (**C**) Schematic representation of HA-Glut4-GFP construct showing the exofacial HA epitope and the GFP tag at the C-terminal region. (**D**) Representative confocal microscopy images showing cell surface staining of tagged GLUT4 in response to stimulation with 100 nM of insulin in C2C12 cells. (**E**) Cellular localization of HA-Glut4-GFP in cells transfected with pCMV and DNAJB3 at baseline (a,b and c versus g,h and i) and after stimulation with 100 nM of insulin (d,e and f versus j,k and l). The images were captured using the tile scanning method; and each image represents 25 adjacent and overlapping fields acquired with a 40X objective. The quantification was done on individual cells and for each condition; we analyzed at least 100 cells. (**F**) Representative image illustrating the plasma membrane localization of HA-Glut4-GFP in C2C12 cotransfected DNAJB3 in the presence of insulin. (**G**) The surface-to-total Glut4 ratio (HA/GFP) at baseline and after insulin simulation was determined by quantitative immunofluorescence and presented as percent change. **P < 0.01.

Glut1 and Glut4 transporters have a central role in basal and insulin-mediated glucose uptake by the skeletal muscle, respectively^30,31^. To determine whether the observed increase in glucose uptake by DNAJB3 is due to increased expression of Glut transporters, we measured the expression levels of both Glut4 and Glut1 in C2C12 myoblasts transfected with either pCMV-DNAJB3 or pCMV. Data displayed in Fig. 6 did not reveal any change in the expression of Glut4 mRNA (Fig. 6A) and protein (Fig. 6B). By contrast, a significant increase in the levels of Glut1 mRNA was observed in cells overexpressing DNAJB3 (P < 0.001; Fig. 6A). This observation, which ruled out the direct effect of DNAJB3 on controlling the endogenous expression of Glut4 expression in C2C12 cells, prompted us to investigate the effect of DNAJB3 on its translocation to the plasma membrane. For this purpose, cells were transfected with pHA-Glut4-GFP (Fig. 6C) and stimulated with insulin and the cellular localization of Glut4 was visualized by confocal microscopy. As expected, the anti HA antibody labels only the Glut4 on the plasma membrane while the GFP reflect the total Glut4 expression (cell surface and intracellular) (Fig. 6D). We next co-transfected cells with pHA-Glut4-GFP and either pCMV or DNAJB3 and monitored Glut4 translocation by immunofluorescence. Accordingly, we observed a marked increase in both basal (Fig. 6E,g–i) and insulin-stimulated (Fig. 6E,j–l,F) Glut4 translocation in DNAJB3 transfected cells as compared to pCMV control both at basal level (Fig. 6E,a–c) and after insulin stimulation (Fig. 6E,d–f). Quantification of the surface-to-total Glut4 ratio (HA/GFP) revealed a 38% of the Glut4 pool is localized to plasma membrane at steady state (pCMV expression). Upon expression of DNAJB3, the surface Glut4 pool is enriched to 48% (P < 0.01; Fig. 6G). In response to insulin, the Glut4 surface pool is increased to 52% in pCMV transfected cells and to 67% in cells overexpressing DNAJB3 (P < 0.01; Fig. 6G).

## Discussion

The aim of the current investigation was to evaluate whether DNAJB3 has a role in modulating metabolic stress and its relationship to glucose metabolism. We demonstrate that DNAJB3: 1- Abrogated both JNK1 and IKKβ pathways in functional assays, 2- Suppressed TNF-α-mediated IL-6 promoter activation and mRNA expression; 3- Reduced ER and oxidative stress and, 4- Enhanced glucose uptake and elicited Glut4 translocation. Altogether, our data provide for the first time a compelling evidence for a novel role of DNAJB3 in modulating metabolic stress; a prerequisite step that leads to IR and type 2 diabetes.

Our interest to elucidate the pathophysiological role of DNAJB3 in glucose metabolism came from our initial observations that the levels of DNAJB3 are reduced in adipose tissue obtained from obese and diabetic subjects and they correlate with increased P-JNK1, enhanced inflammation and ER stress^11,23,32^. More importantly, we showed that physical exercise training restored the normal expression of DNAJB3 while decreasing P-JNK1, inflammatory and ER stress responses^11,32^. Interestingly, the decrease in DNAJB3 levels was more pronounced in obese-diabetic patients as compared to obese non-diabetic subjects^23^. We also reported that DNAJB3 interacts with JNK1 and IKKβ in co-immunoprecipitation assays^23^ and attenuates the activation of JNK in response to palmitate^11^. All these observations suggest a protective role of DNAJB3 against obesity associated metabolic stress.

One of the fascinating pathways that are activated under metabolic stress conditions is the JNK1 kinase pathway, which is known to interfere with insulin signal transduction. The role of activated JNK in phosphorylating IRS-1 substrate at the inhibitory serine 307 residue (IRS-1^S307^) and thus, converting it to an inactive substrate is well established^14^. Recent findings from our group indicate that beside the role of DNAJB3 in attenuating the activation of JNK1, it significantly reduces the phosphorylation of IRS-1^S307^ in response to palmitate while promoting the AKT survival pathway as monitored by increased phosphorylation of AKT protein in HEK-293 cells and 3T3-L1 adipocytes^23^. In addition to its effect on IRS-1, JNK1 plays a fundamental role in modulating gene expression by activating an array of transcription factors and other nuclear proteins involved in apoptosis, inflammation, DNA repair, mRNA stability and development^33,34^. In the current investigation, we provide evidence that DNAJB3 acts as a natural inhibitor of JNK-1 as it abrogates its ability to modulate gene transcription using functional assays, confirming thus our previous demonstration that DNAJB3 binds to JNK1 and reduces its activation in response to palmitate and PMA stressors (Fig. 1). Our results are similar to those reported for HSP-72 in which they showed that all the interventions that lead to the induction of HSP-72 expression are associated with impairment of JNK phosphorylation with concomitant improvement of clinical outcomes in humans and animal models of obesity, IR and type 2 diabetes^11,23^. The nature of such JNK-downstream target genes, their specific role in metabolic stress and the possible modulation of their expression remain to be elucidated using biochemical, cellular and systems biology approaches. This was one of the limitations of the current investigation.

Besides JNK1, a large body of evidence indicates that pathological activation of the IKKβ kinase has detrimental consequence on insulin signaling and glucose metabolism. IKKβ is also an inhibitor of IRS-1 substrate as it phosphorylates its serine 307 residue^15^. IKKβ is a master upstream kinase that activates the canonical pathway of NF-κB^35^. Once activated, it turns on a complex transcription program driven by NF-κB that leads to inappropriate expression and release of an array of inflammatory mediators including cytokines, chemokines, metalloproteases and growth factors^35^. Using a κB-dependent luciferase assay, we show in this current investigation for the first time that both PMA- and TNF-α mediated activation of IKKβ/NF-κB axis are markedly suppressed when DNAJB3 is overexpressed (Fig. 2). To validate further the role of DNAJB3 in modulating the activity of IKKβ kinase in a biologically relevant context, we transfected cells with the luciferase reporter system driven by the entire IL-6 promoter^25^. As expected, we found a marked decrease in the transactivation of IL-6 promoter in response to TNF-α when DNAJB3 is overexpressed (Fig. 2B). These data are consistent with our previous immunoprecipitation data in which we showed that DNAJB3 and IKKβ are part of a large multi-protein complex^11^. In the setting of obesity, the decrease in the expression of DNAJB3 has been shown previously by our group to be concomitant with increased expression of IL-6 mRNA, however it was not clear whether a decrease of DNAJB3 was due to increased expression of IL-6 or vice-versa (i.e., cause/effect relationship). In this investigation, we provide evidence for a functional role of DNAJB3 in reducing the endogenous expression of IL-6 mRNA both in C2C12 (Fig. 3A) and 3T3-L1 (Fig. 3B). These findings could also explain, at least in part, the paradox between DNAJB3 and IL-6 levels in obese subjects. Our results showing anti-inflammatory activity of DNAJB3 are in line with the previous findings on HSP-25/27, another heat shock protein that was shown to bind to IKKβ and inhibits its activity and thereby, improving insulin signaling in skeletal muscle from high fat fed rats^20^. HSP-72 is one of the best-studied chaperones among all the HSPs in relationship to metabolic diseases. Its role in conferring protection against metabolic defects leading to IR and type 2 diabetes in part by reducing the inflammation is extensively reported^36–38^. However, we failed to demonstrate a role of HSP-72 in attenuating the expression of IL-6 mRNA in C2C12 cells under our experimental conditions (Fig. 3A).

Another important aspect in this study is the effect of DNAJB3 on glucose metabolism in skeletal muscle C2C12 as well as the molecular and biochemical determinants mediating such effect. Glucose transport into muscle and fat cells is an important step in insulin action and is critical for the maintenance of glucose homeostasis^39^. We have shown previously that overexpression of DNAJB3 in 3T3-L1 adipocytes resulted in enhanced glucose uptake^23^. In the same study, we showed that DNAJB3 has a positive impact on improving insulin signaling as it prevents IRS-1^S307^ phosphorylation while promoting its phosphorylation at tyrosine 612 (IRS-1^Y612^)^23^. In the current investigation, we used two complementary approaches to investigate the specific effect of DNAJB3 on glucose uptake, namely by increasing and knocking down its expression. Accordingly, we provide strong evidence that DNAJB3 enhances both basal and insulin-stimulated glucose uptake in C2C12 cells (Fig. 5). Indeed, we observed a significant increase in glucose uptake in cells overexpressing DNAJB3 that was independent of insulin action (Fig. 5D). An additive effect of insulin on glucose uptake was observed in C2C12 cells overexpressing DNAJB3 (Fig. 5D). In agreement with this, knocking down the expression of DNAJB3 by silencing RNA blunted the glucose uptake in response to insulin (Fig. 5H). In HepG2 however, DNAJB3 increases only the basal glucose uptake (Fig. 5F).

**Figure 7:**
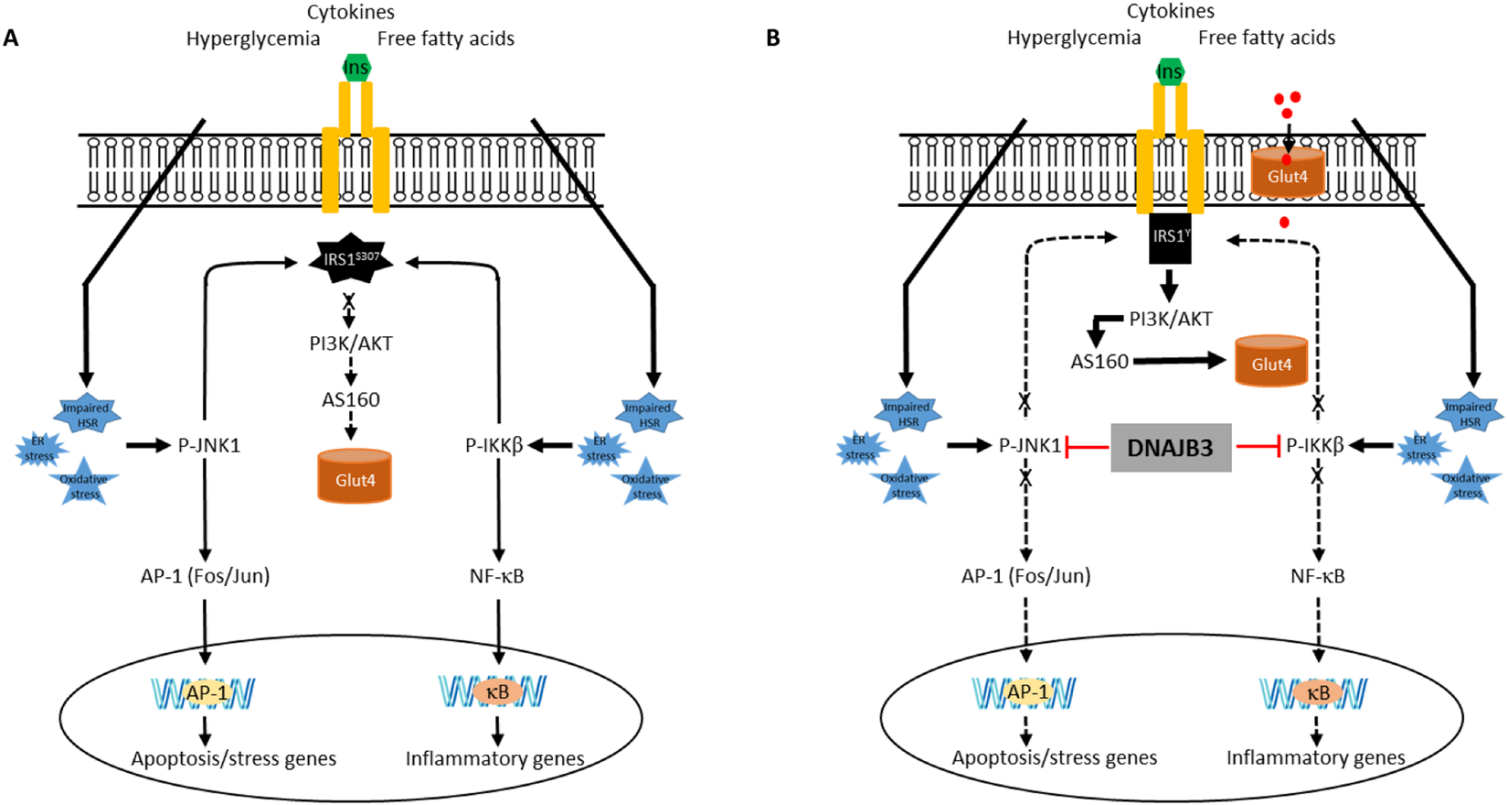
Schematic representation for the role of DNAJB3 in mitigating metabolic stress and improving glucose uptake.

Finally, we investigated the mechanism underlying the DNAJB3-mediated glucose uptake enhancement. In the skeletal muscle, Glut1 and Glut4 have a central role in basal and insulin-mediated glucose mobilization, respectively^30,31^. The observed effect of DNAJB3 on basal glucose uptake is consistent with the finding that DNAJB3 stimulates the expression of Glut1 (Fig. 6A). Insulin elicits its metabolic action by activating multiple signaling cascades in metabolically relevant sites. Of these, the activation of phosphatidylinositol-3-kinase (PI-3K), Akt and its substrate 160 (AS160) are critically involved in insulin-mediated Glut4 translocation and glucose uptake in 3T3-L1 adipocytes and skeletal muscle^40,41^. In human subjects, Akt and AS160 phosphorylation are impaired in skeletal muscle obtained from insulin-resistant patients^42^ as well as upon TNF-α stimulation^43^. Interestingly, the level P-AKT and P-AS160 were significantly increased in 3T3-L1 adipocytes overexpressing DNAJB3^23^. Data presented in the current study indicates that DNAJB3 elicits both basal and insulin-stimulated Glut4 translocation in C2C12 (Fig. 6). These results provide novel insights into the regulatory mechanism by which DNAJB3 stimulates glucose uptake. On the light of the current and previous findings^23^, we propose a model by which DNAJB3 orchestrates its protective effects as illustrated in Fig. 7. As indicated, excessive accumulation of free fatty acids, chronic hyperglycemia and inflammatory mediators lead to the persistent ER stress, oxidative stress and impaired expression of the HSR. This toxic environment will lead to the activation of JNK-1 and IKKβ kinases that target the IRS-1 and convert it to poor substrate of the insulin receptor and ultimately blocking the PI-3K/AKT pathway. At the nuclear level, the activation of JNK-1 and IKKβ leads to the activation of at least two transcriptional programs orchestrated by NF-κB and AP-1 transcription leading thus, to the inappropriate expression and/or release of inflammatory mediators and stress and apoptosis genes (Fig. 7A). Overexpression DNAJB3 prevents the activation of JNK-1 and IKKβ kinases and thereby favoring the PI-3K/AKT pathway that leads to Glut4 translocation as well attenuating the transcriptional programs driven by NF-κB and AP-1 (Fig. 7B).

Our findings on the novel role DNAJB3 in mitigating metabolic stress and improving glucose metabolism raised questions that were not addressed in this current investigation and they deserve consideration for future follow-up studies. For instance, are DNAJB3 KO animals more prone to IR and diabetes? Does DNAJB3 have an effect on controlling the expression of the *in vivo* downstream target genes of JNK-1 and IKKβ? How does DNAJB3 promote basal Glut4 translocation and whether it is specific for Glut4 only?

In summary, we show for the first time that DNAJB3 has a protective role in mitigating metabolic stress by binding to JNK1 and IKKβ enzymes and abrogating their activation in response to harmful stressors. DNAJB3 has also a positive role in improving glucose uptake at least in part by enhancing Glut4 translocation to the plasma membrane in C2C12. Altogether, they suggest a physiological role of DNAJB3 in glucose metabolism and insulin signaling. Identifying small molecules that induce the expression DNAJB3 or recapitulate its function could be leveraged as a possible novel strategy for the control and management of metabolic defects leading to IR and type 2 diabetes.

## Supplementary information

Supplementary Information

## References

[1] Defronzo RA (2009). Banting Lecture. From the triumvirate to the ominous octet: a new paradigm for the treatment of type 2 diabetes mellitus. Diabetes.

[2] DeFronzo RA (2015). Type 2 diabetes mellitus. Nature reviews. Disease primers.

[3] Upadhyay J, Farr O, Perakakis N, Ghaly W, Mantzoros C (2018). Obesity as a Disease. The Medical clinics of North America.

[4] Hotamisligil GS (2017). Inflammation, metaflammation and immunometabolic disorders. Nature.

[5] Poitout V, Robertson RP (2008). Glucolipotoxicity: fuel excess and beta-cell dysfunction. Endocrine reviews.

[6] Houstis N, Rosen ED, Lander ES (2006). Reactive oxygen species have a causal role in multiple forms of insulin resistance. Nature.

[7] Szendroedi J, Phielix E, Roden M (2011). The role of mitochondria in insulin resistance and type 2 diabetes mellitus. Nature reviews. Endocrinology.

[8] Engin F, Hotamisligil GS (2010). Restoring endoplasmic reticulum function by chemical chaperones: an emerging therapeutic approach for metabolic diseases. Diabetes, obesity & metabolism.

[9] Pirola L, Ferraz JC (2017). Role of pro- and anti-inflammatory phenomena in the physiopathology of type 2 diabetes and obesity. World journal of biological chemistry.

[10] Picu, A. *et al*. Markers of Oxidative Stress and Antioxidant Defense in Romanian Patients with Type 2 Diabetes Mellitus and Obesity. *Molecules (Basel*, *Switzerland)* **22** (2017). 10.3390/molecules22050714 PMC6154306 28468307

[11] Abubaker J (2013). DNAJB3/HSP-40 cochaperone is downregulated in obese humans and is restored by physical exercise. PloS one.

[12] Rogers RS (2016). Deficiency in the Heat Stress Response Could Underlie Susceptibility to Metabolic Disease. Diabetes.

[13] Wellen KE, Hotamisligil GS (2005). Inflammation, stress, and diabetes. The Journal of clinical investigation.

[14] Aguirre V, Uchida T, Yenush L, Davis R, White MF (2000). The c-Jun NH(2)-terminal kinase promotes insulin resistance during association with insulin receptor substrate-1 and phosphorylation of Ser(307). The Journal of biological chemistry.

[15] Gao Z (2002). Serine phosphorylation of insulin receptor substrate 1 by inhibitor kappa B kinase complex. The Journal of biological chemistry.

[16] Saibil H (2013). Chaperone machines for protein folding, unfolding and disaggregation. Nature reviews. Molecular cell biology.

[17] Park KJ, Gaynor RB, Kwak YT (2003). Heat shock protein 27 association with the I kappa B kinase complex regulates tumor necrosis factor alpha-induced NF-kappa B activation. The Journal of biological chemistry.

[18] Park HS, Lee JS, Huh SH, Seo JS, Choi EJ (2001). Hsp72 functions as a natural inhibitory protein of c-Jun N-terminal kinase. The EMBO journal.

[19] Simar D, Jacques A, Caillaud C (2012). Heat shock proteins induction reduces stress kinases activation, potentially improving insulin signalling in monocytes from obese subjects. Cell stress & chaperones.

[20] Gupte AA, Bomhoff GL, Swerdlow RH, Geiger PC (2009). Heat treatment improves glucose tolerance and prevents skeletal muscle insulin resistance in rats fed a high-fat diet. Diabetes.

[21] Chung J (2008). HSP72 protects against obesity-induced insulin resistance. Proceedings of the National Academy of Sciences of the United States of America.

[22] Hooper PL (1999). Hot-tub therapy for type 2 diabetes mellitus. The New England journal of medicine.

[23] Abu-Farha M (2015). DNAJB3/HSP-40 cochaperone improves insulin signaling and enhances glucose uptake *in vitro* through JNK repression. Scientific reports.

[24] Blot V, McGraw TE (2006). GLUT4 is internalized by a cholesterol-dependent nystatin-sensitive mechanism inhibited by insulin. The EMBO journal.

[25] Eickelberg O (1999). Transforming growth factor-beta1 induces interleukin-6 expression via activating protein-1 consisting of JunD homodimers in primary human lung fibroblasts. The Journal of biological chemistry.

[26] Fahmi H (2001). Peroxisome proliferator–activated receptor gamma activators inhibit interleukin-1beta-induced nitric oxide and matrix metalloproteinase 13 production in human chondrocytes. Arthritis and rheumatism.

[27] Wang C (2018). Glutamine Enhances the Hypoglycemic Effect of Insulin in L6 Cells via Phosphatidylinositol-3-Kinase (PI3K)/Protein Kinase B (AKT)/Glucose Transporter 4 (GLUT4) Signaling Pathway. Medical science monitor: international medical journal of experimental and clinical research.

[28] Derijard B (1994). JNK1: a protein kinase stimulated by UV light and Ha-Ras that binds and phosphorylates the c-Jun activation domain. Cell.

[29] Wang Y (2000). Activation of ATF6 and an ATF6 DNA binding site by the endoplasmic reticulum stress response. The Journal of biological chemistry.

[30] Bell GI (1991). Lilly lecture 1990. Molecular defects in diabetes mellitus. Diabetes.

[31] Thorens B, Mueckler M (2010). Glucose transporters in the 21st Century. American journal of physiology. Endocrinology and metabolism.

[32] Khadir A (2016). Physical exercise alleviates ER stress in obese humans through reduction in the expression and release of GRP78 chaperone. Metabolism: clinical and experimental.

[33] Ventura JJ, Kennedy NJ, Lamb JA, Flavell RA, Davis RJ (2003). c-Jun NH(2)-terminal kinase is essential for the regulation of AP-1 by tumor necrosis factor. Molecular and cellular biology.

[34] Hotamisligil, G. S. & Davis, R. J. Cell Signaling and Stress Responses. *Cold Spring Harbor perspectives in biology* **8** (2016). 10.1101/cshperspect.a006072 PMC5046695 27698029

[35] Dulloo AG, Jacquet J, Solinas G, Montani JP, Schutz Y (2010). Body composition phenotypes in pathways to obesity and the metabolic syndrome. International journal of obesity (2005).

[36] Henstridge DC (2014). Activating HSP72 in rodent skeletal muscle increases mitochondrial number and oxidative capacity and decreases insulin resistance. Diabetes.

[37] Hooper PL, Hooper PL (2009). Inflammation, heat shock proteins, and type 2 diabetes. Cell stress & chaperones.

[38] Kondo T (2010). Heat shock treatment with mild electrical stimulation safely reduced inflammatory markers in healthy male subjects. Obesity research & clinical practice.

[39] Bryant NJ, Govers R, James DE (2002). Regulated transport of the glucose transporter GLUT4. Nature reviews. Molecular cell biology.

[40] Kane S (2002). A method to identify serine kinase substrates. Akt phosphorylates a novel adipocyte protein with a Rab GTPase-activating protein (GAP) domain. The Journal of biological chemistry.

[41] Larance M (2005). Characterization of the role of the Rab GTPase-activating protein AS160 in insulin-regulated GLUT4 trafficking. The Journal of biological chemistry.

[42] Karlsson HK (2005). Insulin-stimulated phosphorylation of the Akt substrate AS160 is impaired in skeletal muscle of type 2 diabetic subjects. Diabetes.

[43] Plomgaard P (2005). Tumor necrosis factor-alpha induces skeletal muscle insulin resistance in healthy human subjects via inhibition of Akt substrate 160 phosphorylation. Diabetes.

