# Supplementary material for: DNAJB3 attenuates metabolic stress and promotes glucose uptake by eliciting Glut4 translocation
Source: Sci Rep. 2019 Mar 18;9:4772. doi: 10.1038/s41598-019-41244-8 (PMC6423224; doi:10.1038/s41598-019-41244-8)
Supplement: Supplementary file 1 — Supplementary Information [file 41598_2019_41244_MOESM1_ESM.docx]

**Supplementary information**

**DNAJB3 attenuates metabolic stress and promotes glucose uptake by eliciting Glut4 translocation**

Abdelilah Arredouani^1#^, Abdoulaye Diane^1#^, Namat Khattab^1^, Ilham Bensmail^1^, Imad Aoude^1^, Mohamed Chikri^2^, Ramzi Mohammad^3-4^, Abdul Badi Abou Samra^5^ & Mohammed Dehbi^1*^

^1^Qatar Biomedical Research Institute, Hamad Bin Khalifa University, Doha, Qatar

^2^Faculty of Medicine & Pharmacy, University Sidi Mohamed Ben Abdellah, Fes, Morocco

^3^The Interim Translational Research Institute, Hamad Medical Corporation, Doha, Qatar

^4^Karmanos Karmanos Cancer Institute, Department Of Oncology, Wayne State University, Detroit, MI, USA

^5^Qatar Metabolic Institute, Department of Internal Medicine, Hamad Medical Corporation, Doha, Qatar

^#:^ These authors contributed equally to this work.

**Supplementary Figure S1**


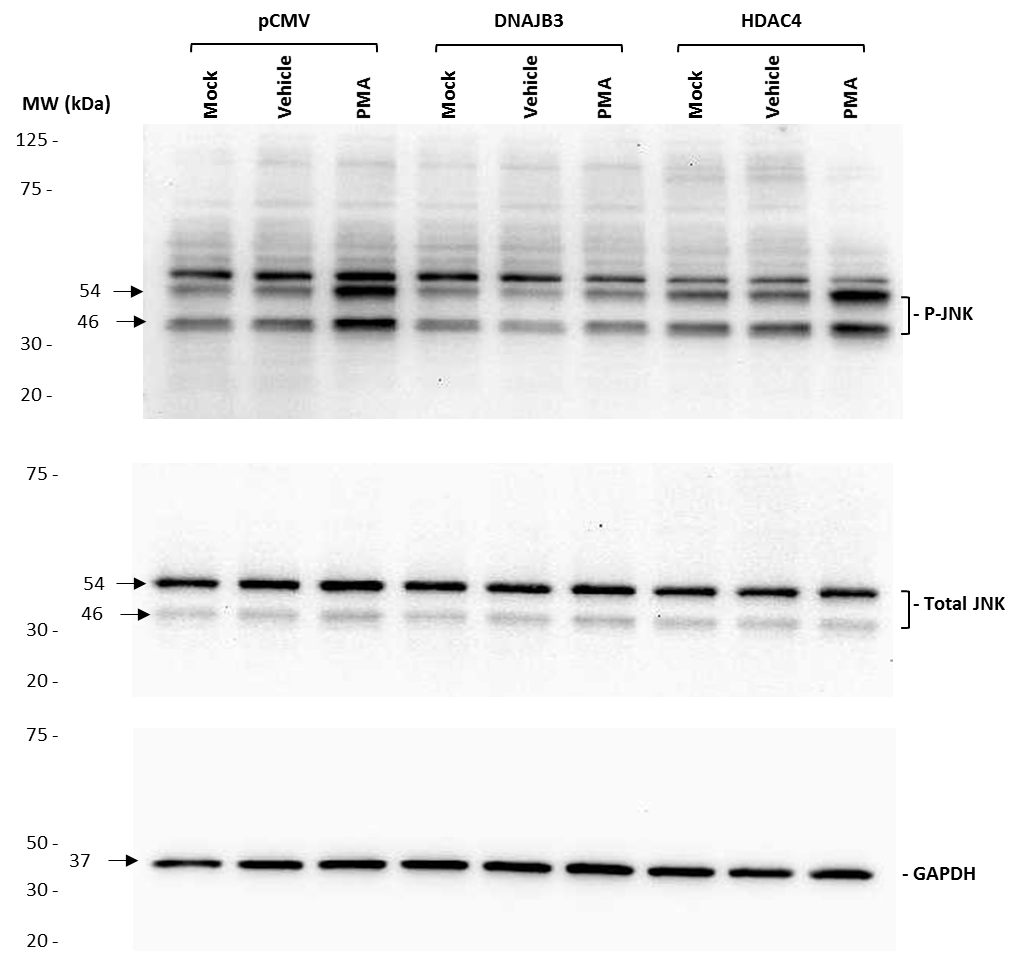


**Supplementary Figure S1:** Transient overexpression of DNAJB3 in HEK-293 cells prevents the phosphorylation of JNK (P-JNK) in response to phorbol myristate acetate (PMA) as compared to pCMV and pCMV-HDAC4. Total JNK and GAPDH were used as internal controls to monitor for protein loading differences. After determining the levels of P-JNK, the same membrane was stripped and probed with antibody against total JNK.

**Supplementary Figure S2**

**
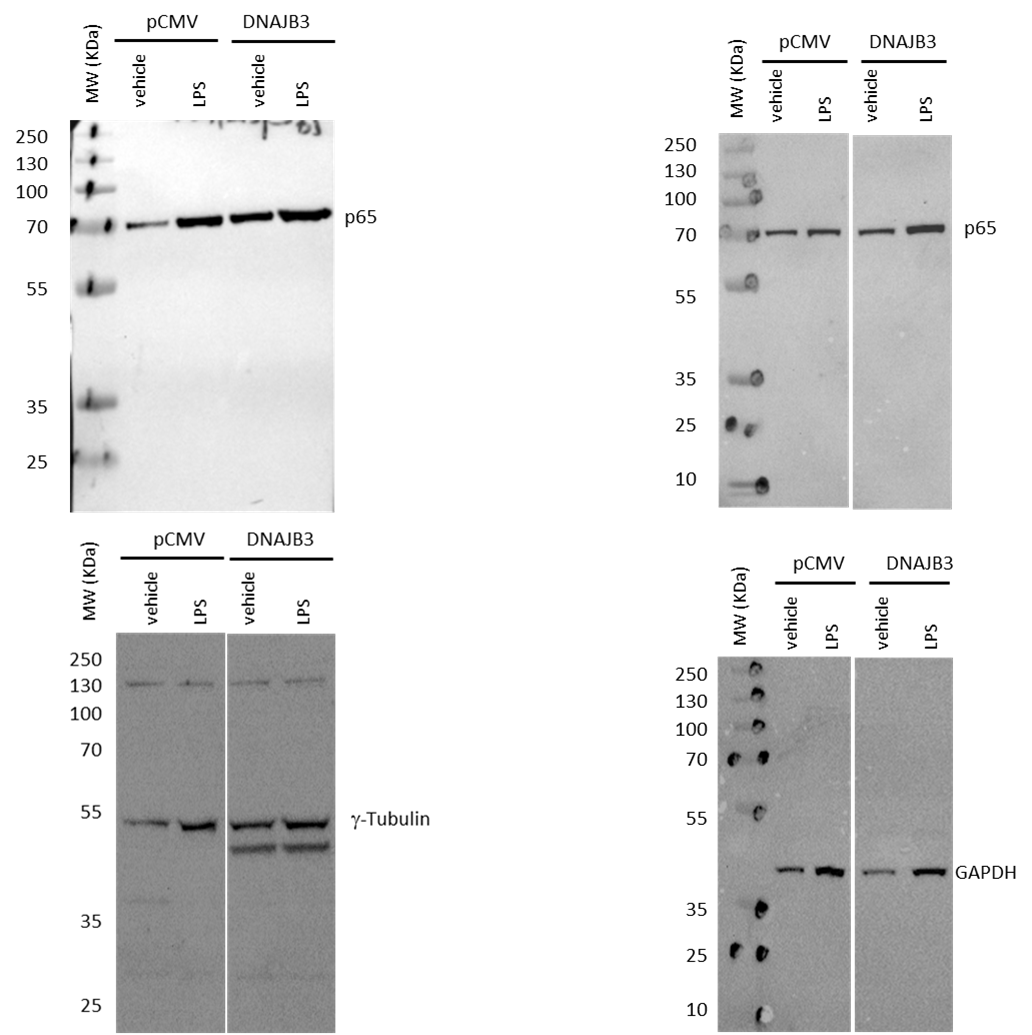
**

**Supplementary Figure S2.** Overexpression of DNAJB3 in C2C12 myoblasts reduces the translocation of p65 NF-κB to the nucleus in response to LPS treatment. γ-Tubulin and GAPDH were used as internal controls for nuclear and cytoplasmic extracts, respectively.

**Supplementary Figure S3**

**
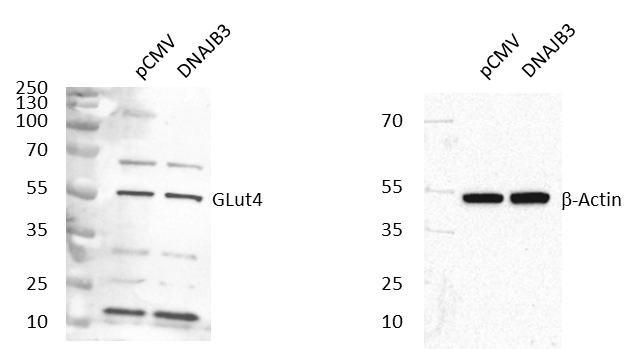
**

**Supplementary Figure S3.** DNAJB3 overexpression has no effect on the endogenous expression of Glut4 in C2C12 cells. β-Actin was used as internal control to correct for loading efficiency.
